# Effect of vaccines against pancreas disease in farmed Atlantic salmon

**DOI:** 10.1111/jfd.13505

**Published:** 2021-08-17

**Authors:** Magnus Vikan Røsæg, Ragnar Thorarinsson, Arnfinn Aunsmo

**Affiliations:** ^1^ SalMar Farming AS Trondheim Norway; ^2^ Elanco Animal Health Bergen Norway; ^3^ Faculty of Veterinary Medicine University of Life Sciences Oslo Norway; ^4^ Present address: Laxar fiskeldi ehf Eskifjörður Iceland

**Keywords:** Atlantic salmon, pancreas disease, SAV, vaccine

## Abstract

Pancreas disease (PD) caused by salmonid alphavirus (SAV) continues to negatively impact salmon farming. To assess the effect on growth and mortality of three vaccines against PD, two controlled field designs were employed: one controlled field study with individual marked fish (PIT tag) assessing three PD vaccines and three controls groups, and a second controlled field study with group marked fish (Maxilla) comparing two PD vaccines against controls. In addition, a descriptive study using whole cages compared fish immunized with two different PD vaccines against controls.

The target populations experienced a natural PD outbreak where both SAV 2 and SAV 3 were identified. Only one of the PD vaccines provided statistically significant improvements in harvest weight of 0.43 kg (CI: 0.29–0.57) and 0.51 kg (CI: 0.36–0.65) compared with the control in the PIT tag and the Maxilla study, respectively. In the latter, a significant reduction in mortality of 1.31 (CI:0.8–1.8) per cent points was registered for the same vaccine compared with controls. These results aligned with the growth and PD‐specific mortality registered in the descriptive Cage study. The data in this study show a difference in the efficacy of PD vaccines in farmed Atlantic salmon.

## 1 INTRODUCTION

Since first described in 1976, pancreas disease (PD) has represented an economically important viral disease affecting seawater‐reared Atlantic salmon (*Salmo salar*) in Ireland, UK and Norway (Jansen et al., 2017; McLoughlin & Graham, 2007; Munro et al., 1984). The aetiological agent, formally named salmon pancreas disease virus (SPDV) and commonly denoted as salmonid alphavirus (SAV), partly because the virus also infects other salmonid species (Jansen et al., 2017; Lewisch et al., 2018; Villoing et al., 2000).

Histopathological analysis of PD reveals acute necrosis or absence of exocrine pancreas, cardiomyocytic necrosis with subsequent inflammation of heart tissue, as well as necrosis and inflammation of red and white skeletal muscle (McLoughlin & Graham, 2007). The disease usually manifests as a sudden drop in appetite followed by lethargy and often increased mortality. The most pronounced consequences are reduced fish welfare, reduced growth, increased feed conversion ratio, mortality and reduced harvest quality (Jansen et al., 2017; McLoughlin & Graham, 2007). The direct cost of an outbreak with SAV, subtype 3, in 2013 in Norway was estimated to average NOK 55.4 million on a sea site starting with 1 million smolts (Pettersen et al., 2015).

Salmonid alphavirus has been divided into six subtypes, SAV 1 to SAV 6, based on phylogenetic analysis of the glycoprotein E2 and the non‐structural protein nsP3 (Fringuelli et al., 2008), although whole‐genome sequencing of a SAV isolated from Ballan wrasse suggests a new subtype (SAV 7) (Tighe et al., 2020). An earlier cross‐neutralization study demonstrated close serological relatedness among five of the six subtypes currently known with the possible exception of SAV 6 (Graham et al., 2014). These results suggest that a vaccine based on a single SAV subtype might protect against PD caused by a different subtype. There is strong support of a difference in virulence between subtypes, where SAV 1 and SAV 3 are considered to be most virulent (Graham et al., 2011; Jansen et al., 2015; Johansen et al., 2015). All subtypes except SAV 3 have been detected on the British Isles, while in Norway there are currently two subtypes, SAV 3 and SAV 2, which are largely limited to separate enzootic zones (Anonymous, 2017; Hjortaas et al., 2013, 2016; Jansen et al., 2017).

Numerous efficacy studies using different types of vaccines have demonstrated significant level of protection against PD in experimental settings (Chang et al., 2017; Hikke et al., 2014; Karlsen et al., 2012; López‐Dóriga et al., 2001; Skjold et al., 2016; Thim et al., 2012; Thorarinsson et al., 2021; Xu et al., 2012). An earlier cohort study performed in the enzootic SAV 3 area in Norway showed reduction in occurrence and severity of PD outbreaks provided by the PD vaccine available at that time (Bang Jensen et al., 2012). Despite extensive vaccination against PD in this area, the disease continued to cause serious welfare concerns and economic losses (Pettersen et al., 2015). In 2012, an experimental PD vaccine was shown to provide significantly improved protection against PD in both laboratory and field experiments compared with the commercially available PD vaccine (Karlsen et al., 2012).

In this paper, we report the results from two controlled field studies and one descriptive Cage study using 2018 smolt generation. The objective of the study was to investigate and compare the effects of PD vaccines focusing mainly on recently licensed vaccines in Norway. The main outcome variables in this study were mortality and growth.

## 2 MATERIAL AND METHODS

### 2.1 Target population

**FIGURE 1:**
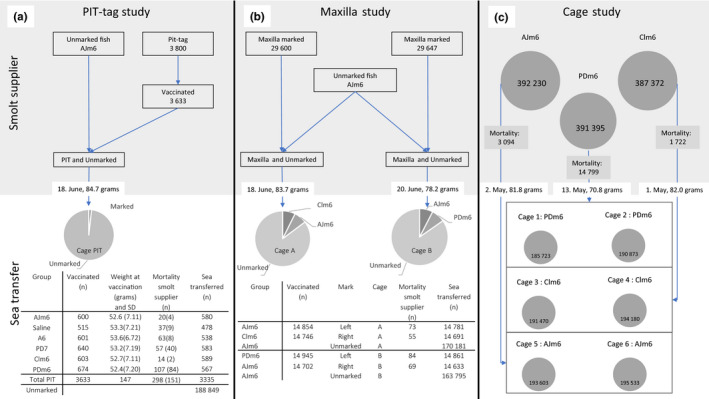
Schematic layout of the target population in the three studies, PIT‐tag study (a), Maxilla study (b) and Cage study (c). A; Shows the flow of the fish groups from marking until sea transfer. The table displays the vaccine groups and number of fish vaccinated per group. Also shown is mean weight at vaccination including one standard deviation, overall mortality count in marked fish at the smolt facility with the number fish registered dead in connection to vaccination in brackets and the number of fish sea transferred. B: Shows the flow of the fish groups from after vaccination and marking to sea transfer. The table displays the number of fish vaccinated in each vaccine group, the mark, mortality at the smolt facility and the number of fish transferred to the two cages. C: Shows the flow of the fish in the six cages including number of fish from vaccination, vaccine group, mortality at the smolt facility and the starting number of fish in each cage

The clinical field trial described in this paper involves three studies in sea water employing different experimental designs: two controlled field studies hereinafter referred to as the PIT tag and the Maxilla studies, and a descriptive Cage study (Cage study). All three studies were carried out using 157‐metre circumference net cages with commercially reared Atlantic salmon. The PIT‐tag study included a small proportion of fish individually tagged with passive integrated transponders (PIT) tags reared within a single cage (PIT). The Maxilla study included two cages (A and B) with a proportion fish physically marked by removing right or left maxilla. A total of 6 cages (Cages 1–6) were used in the Cage study holding fish vaccinated with three different vaccine combinations. The fish in the above studies were reared in total nine cages, hereinafter referred to as the “target population.” The target population was reared from ova (AquaGen strain) at the same hatchery (Site no. 13958, SalMar Settefisk AS, Follafoss) from August 2017 until transfer to sea water the spring of 2018 (S1 smolts). The fish and freshwater tanks were tended and monitored daily according to the smolt facilities standard operating procedures. Layout and number of fish transferred to sea are displayed in Figure 1.

The Maxilla and PIT‐tag study involved marking of fish that was approved by the Norwegian Animal Research Authority (FOTS ID: 14,067) prior to study start. Only healthy, intact and sexually immature fish without visual deformities heavier than 35 grams were included in the study.

### 2.2 Vaccines and vaccination

All vaccines used in this study are commercially available in Norway. The vaccine brand names, their route of administration, the infectious agents they protect against, dosage and assigned study group identifications are outlined in Table 1. The fish were starved for 2–4 days and then anaesthetized with tricaine methanesulphonate (Tricaine, Pharmaq) prior to PIT‐tag marking (PIT‐tag study) and vaccination. Number of day degrees (dd) from vaccination to sea transfer ranged from 653 to 757 dd, thus exceeding the minimum as recommended in the summary of product characteristics (SPC) for the vaccines used in the study.

**TABLE 1:** Overview of vaccine group identity and dosage used in this study

Vaccine group	Vaccine name	Dose (ml) and route	Group type	Included in
AJm6	ALPHA JECT micro^®^ 6^1^	0.05 i.p.	Baseline	PIT‐tag, Maxilla, Cage
Clm6	ALPHA JECT micro^®^ 6^1^	0.05 i.p.	Test	PIT‐tag, Maxilla, Cage
	Clynav^2^	0.05 i.m.		
PDm6	ALPHA JECT micro^®^ 6^1^	0.05 i.p.	Test	PIT‐tag, Maxilla, Cage
	ALPHA JECT micro^®^ 1 PD^3^	0.05 i.p.		
Saline	Saline^6^	0.05 i.p.	Control	PIT
PD7	Aquavac PD 7 vet.^5^	0.1 i.p.	Test	PIT
A6	Aquavac 6 vet.^4^	0.1 i.p.	Control	PIT

Note

^1, 4 and 5^ Contain inactivated *Aeromonas salmonicida* subsp. *salmonicida*, *Aliivibrio salmonicida*, *Listonella anguillarum* serotype O1, *L. anguillarum* serotype O2a, *Moritella viscosa* and infectious pancreatic necrosis virus (IPNV). ^1 and 3^ Oil adjuvant vaccine produced by Pharmaq, where ^3^ contains inactivated SPDV. ^2^ A DNA‐based vaccine produced by Elanco Animal Health. For details, see SPC (www.ema.europa.eu/documents/product‐information/clynav‐epar‐product‐information_en.pdf). ^4 and 5^ Oil adjuvant vaccine produced by MSD Animal Health where ^5^ contains inactivated SAV. ^6^ All groups, including Saline, were in addition immunized i.p. with ALPHA DIP ERM (produced by Pharmaq) containing formalin‐inactivated Yersinia ruckeri serotype O1b; the dosage of this component in the Maxilla and Cage studies was 0.02 ml and in the PIT‐tag study diluted with Saline 1:1 to a total dose of 0.05 ml.

### 2.3 PIT‐tag study

In the PIT‐tag study, 3,800 fish were anaesthetized and i.p.‐injected with PIT tags (BIOMark HTP12, preloaded tags), and held in a separate tank until vaccination was completed in two operations 14 and 21 days later. A total of 3,633 fish were vaccinated and assigned to the test groups as described in Figure 1a. After vaccination, tagged fish were mixed with the rest of the fish population in the cage. To secure unintentional bias, the fish were randomly collected using a dip net and vaccinated in blocks of approximately 300 fish per vaccine group. The fish were vaccinated manually to allow for individual fork lengths, weight and group identity to be registered and uploaded into the PIT‐tag database. For vaccine groups Clm6, PDm6 and AJm6, MICOR‐MATIC^®^ (Pharmaq, Norway) twin injector was used for simultaneous i.p. administration of ALPHA JECT micro^®^ 6 and ALPHA DIP ERM Salar. The monovalent PD vaccines in groups Clm6 and PDm6, Clynav and ALPHA JECT micro 1 PD, were administered separately i.m. and i.p., respectively, using a Socorex^®^ Automatic syringe (Socorex Isba SA, Switzerland). For groups PD7 (Aquavac PD7 vet) and A6 (Aquavac 6 vet), ALPHA DIP ERM Salar was administered separately. The fish were adipose fin‐clipped (AFC) immediately after vaccination to allow for additional visual identification of fish assigned to this study. Increased mortality in groups PD7 and PDm6 was noted on the first day of vaccination, probably caused by accidental over‐exposure to the anaesthetic solution for some fish. These fish were replaced reducing PIT‐tagged fish available for the Saline group. The number of fish vaccinated per group, mortality and date of the remaining numbers and stocked into the recipient cage (PIT) are summarized in Figure 1a.

### 2.4 Maxilla study

In the Maxilla study, vaccination, both i.p. and i.m., was performed simultaneously using a fully automated vaccination machine (Skala‐Maskon, Stjordal, Norway) in accordance with established procedures. Batches of approximately 7,500 fish were vaccinated per group at a time to avoid unintentional bias and at reduced speed to enable removal of right or left maxilla using scissors. The remaining of the unmarked fish used to stock these cages were immunized the same week with the same vaccines as group AJm6. Based on the automatic registrations of the vaccination machine, the weights during immunization of all the fish used in the Maxilla study averaged 54.0 grams. The number of fish vaccinated per group, mortality post‐vaccination at the smolt supplier facility followed by fish numbers and transfer dates to the recipient cages A and B are summarized in Figure 1b.

### 2.5 Cage study

The non‐marked fish assigned to the Cage study were vaccinated using automatic equipment as previously explained leading to two sea cages of fish per vaccine groups AJm6, Clm6 and PDm6. The number of fish vaccinated in each of the groups, mortality post‐vaccination at the smolt supplier facility followed by average weights, fish numbers and dates when transferred to each of the 6 cages are shown in Figure 1c.

### 2.6 Management and sampling at the sea sites

The target population was transferred to sea cages located at two neighbouring sites, Reiråklakken (site number: 29116) and Fuglåsen (site number: 31437), in May and June 2018, both located in the enzootic SAV 2 zone. In the PIT‐tag and the Maxilla study, the identity of the groups was not revealed to the production personnel (blinded). The fish in the study were reared and monitored in accordance with Norwegian regulations and the producer's standard operating procedures (SOPs). This entailed monthly inspections of the target population by professional fish health personnel. The producer's SOP for registration of mortality causes followed previously published methodology (Aunsmo, Bruheim, et al., 2008). Moribund and dead fish were screened monthly for presence of SAV RNA using validated RT‐qPCR methodology (PatoGen AS, Ålesund, Norway) as mandated by Norwegian regulations. All samples with detection of SAV RNA were subsequently subtyped based on PatoGen's qPCR assays specific for SAV 2 and SAV 3.

Pancreas disease regulations in Norway aim to maintain SAV 2 and SAV 3 as geographically separated enzootic zones. Consequently, detection of SAV 3 in fish residing in the SAV 2 zone may, based on national PD regulations, result in a stamping out call by the Norwegian Food Safety Authority (NFSA). After detection of SAV 3 at a neighbouring sea site in November 2018, it was decided to apply to the NFSA for permission to move the target population southwards into the SAV 3 zone. This was followed by the first detection of SAV 2 by RT‐qPCR in the target population in December 2018. One month later, additional detection of SAV 3 was confirmed. The transportation to the new sea site was conducted using well boats between 23 January and 3 February 2019 (site number: 32197 and 12268) without interrupting the integrity of the cages included in this study. In September 2019, two cages (5 and 6) were moved to a neighbouring site (site number: 27215) due to maximal biomass restrictions. See supplementary material 1 and 2 for geographical locations of the sea cage sites used in the study and movement of fish.

Clinical PD with a drop in appetite and increased mortality in the target population was registered in May 2019. The following month, on 24 June, fish were collected from the two cages in the Maxilla study with a crowding net and using a dip net. While unmarked fish were returned to the cage, the fish with the right or left maxilla removed were killed using benzocaine (Benzoak vet; ACD Pharmaceuticals). The analysis included RT‐qPCR as described before of heart tissues for detection of SAV RNA, and scoring of abdominal adhesion from 0 to 6 as previously described (Midtlyng et al., 1996) was carried out in a blinded manner by an experienced fish health professional.

### 2.7 Sampling and data registration during harvest

The fish were harvested at Vikenco AS (site number: 27395) during the latter half of 2019. During harvest of the fish from the PIT‐tag study cage, AFC, and thus PIT‐tag marked fish, was collected from the harvest line at the station for quality sorting. Each AFC individual was scanned for PIT‐tag ID followed by registration of fork length, weight, sex (based on gonad after gutting) and removal of the PIT tag. The first 210 fish collected were, in addition, subjected to abdominal adhesion scoring in a blinded manner as described before. After completion of registrations, the vaccine group affiliations were merged with each PIT‐tag ID.

Sampling of fish at the harvest facility from the two cages included in the Maxilla study (with their right or left maxillary removed) was carried out similarly as in the PIT‐tag study. Between 430 and 500, marked fish from each group in each of the two cages were collected from the harvest line and their fork lengths and weights measured and registered in a blinded manner.

## 3 DATA MANAGEMENT AND STATISTICAL ANALYSIS

### 3.1 PIT‐tag study and Maxilla study

Dead marked fish used in the Maxilla and the PIT‐tag study were collected daily and registered. At the smolt supplier, each dead fish with removed adipose fin was frozen for later registration of the PIT‐tag number. At the sea sites, the PIT tag was removed from the marked fish and archived together with the date of death, and later registered. The registrations connected with the PIT‐tag identity including vaccine group, date of vaccination, time of death and the harvest data were recorded and stored in a Microsoft Access based database system (VESO‐Vikan, Namsos, Norway).

### 3.2 Cage study

Collection and compilation of production data from the 6 cages were carried out using production software (Fishtalk, Aquagroup, Norway) and included weights, growth, expected growth, mortality including cause, biological feed conversion ratio (bFCR), number of days not fed and sea lice treatments. The data were thereafter exported to Microsoft Excel (Microsoft Corporation) monthly, and the accumulated dataset was compiled including the data collected in the harvest process. Growth was evaluated using relative growth index (RGI) (Skretting). The RGI is calculated using specific growth rate (SGR) that is adjusted for water temperature and fish weights at any given time point according to the matrix illustrated in supplementary material 3. The RGI depicts growth rate expectation throughout the seawater production phase, thus calculating arbitrary value (index) of 100. Such conditions were applied to the production software to model the expected harvest weights of fish in each of the 6 cages used in the Cage study. This approach enables comparison of growth between fish reared in different cages irrespective of harvest time points using normalized harvest weight based on the average expected harvest weight (RGI). Normalized harvest weight was calculated using the formula below as follows. Achieved harvest weight (X), divided on the expected harvest weight (E(X)) for each cage, multiplied with the average expected harvest weight (E(µ)) in the target population (5.07 kg).

$$Normalized\;harvest\;weight=\frac{X}{E\left(X\right)}\times E\left(\mu \right).$$

### 3.3 Statistical analysis

The data were exported from the PIT‐tag database or entered manually (Maxilla study) into Microsoft Excel. Maximum and minimums values were investigated to control for registration errors. The statistical analysis was carried out using RStudio (RStudio, 2020).

The cut‐off value for detection of the RT‐qPCR analysis was set to a Ct‐value of 37, non‐detects were given the value 38. In the Maxilla study, a non‐parametric Wilcoxon rank‐sum test (*p* < .05) was used to compare levels of viral RNA between the test groups (Clm6 and PDm6) and the control groups (AJm6) in each of the two cages. To evaluate sampling per cent as a proxy for survival of the PIT‐tagged groups and mortality rates in the Maxilla study, a pairwise two‐sample test for equality of proportion with continuity correction was used.

The abdominal adhesion scores were compared applying a non‐parametric approach with pairwise comparison using a Wilcoxon rank‐sum test with a significant threshold of 0.05 (ordinal variable).

To test the effect of vaccine group on harvest weight, an ordinary least‐squares (OLS) regression model was built, for both the PIT‐tag and the Maxilla study. In the PIT‐tag study, sex (female = 0, male = 1) and individual weights at vaccination were included as two variables previously shown to impact harvest weight in an initial model. Harvest weight in both the PIT‐tag and Maxilla study revealed tendency towards left skewed and a bimodal distribution at the left tale. Therefore, after explorative analysis, including histograms of K‐factor. Runts as a categorical variable defined as k‐factor lower than 1 equal to runt (denoted 0 = no and 1 = yes) was included both in the PIT‐tag and in the Maxilla study. Variables were retained in the models if *p* < .05 and increasing the adjusted *R* ^2^. The residuals from the final model were plotted to ascertain normality and homoscedasticity of the data.

## 4 RESULTS

### 4.1 Diagnostics

**FIGURE 2:**
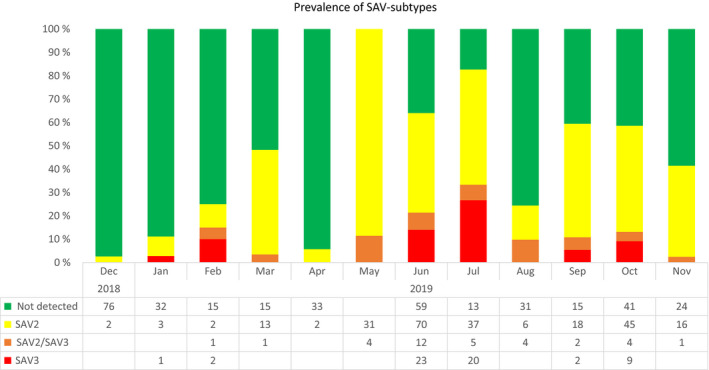
Development of monthly prevalence of SAV‐specific RNA in the target population from December 2018 to harvest. The results include all samples collected (*n* = 694), including dead and moribund fish from the surveillance programme, fish health inspection visits and samples from apparent healthy fish in the Maxilla study sampled in June (see also Figure 6)

In December 2018, the first RT‐qPCR‐positive SAV detection in the target population was identified as SAV 2. Subsequent monthly samples revealed presence of both SAV 2 and SAV 3 throughout the production cycle. The overall prevalence of SAV 2 was higher than SAV 3 counting for 73 and 17% of the samples with detected SAV RNA, respectively. During this infective period, a total of 35 fish, counting for 10% of the samples, with detection of SAV, were found to contain both SAV 2 and SAV 3 (Figure 2).

**FIGURE 3:**
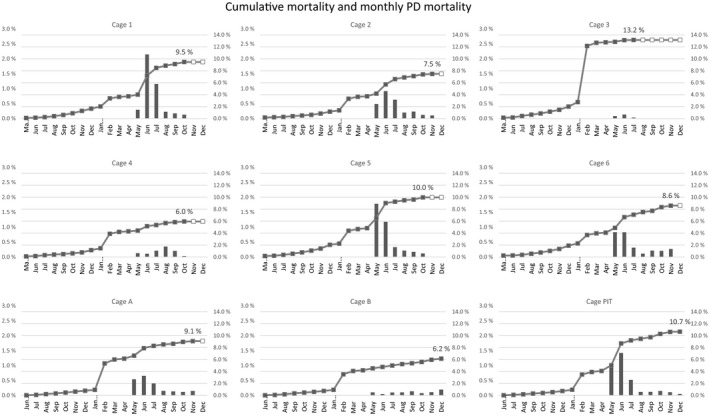
Cumulative mortality (line graph, right axis) and mortality caused by PD (bar graph, left axis) in per cent of monthly mortality registered in the target population. White dots on the line graph indicate that the cage is harvested

A steep increase in mortality occurred in all the study cages subsequent to the well boat transportation to the SAV 3 zone in January and February 2019. The fish in Cage 3 suffered the hardest impact with more than 10% cumulative mortality. Despite presence of SAV detected several months earlier, clinical onset of a PD outbreak did not occur until May, as evaluated by the site manager and the external fish health service. Although most of the mortality caused by PD lasted into July 2019, fish continued to die from the disease until harvest (Figure 3). Average PD‐related mortality counted for one‐fourth (cage level range 1.5%–41.3%) of the total cumulative mortality of 8.9%.

### 4.2 PIT‐tag study

**FIGURE 4:**
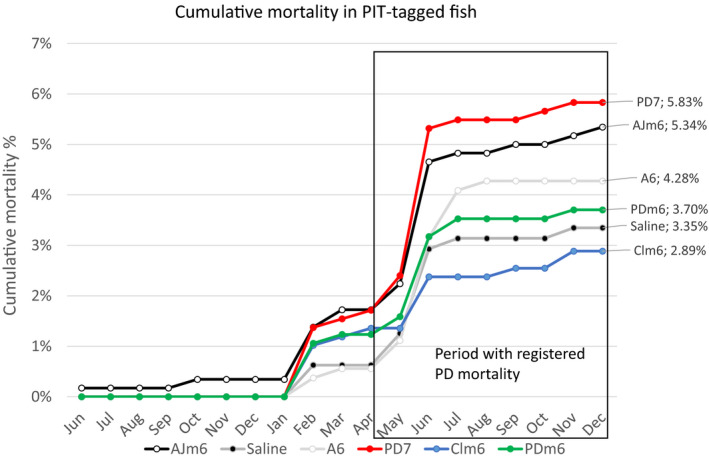
Cumulative mortality, of the PIT‐tag study by vaccine group and month. Box indicates the period of mortality caused by PD

Increase in moribund and dead fish showing clinical signs of PD was registered in May in the PIT‐tag cage. From that time point and until harvest, the mortality caused by PD counted for approximately one‐third (3.3%) of the total cumulative seawater mortality of 10.7% in this cage (Figure 3). The cumulative mortality until harvest ranged from 2.9% to 5.8% in groups Clm6 and PD7, respectively. This result was strongly influenced by difference in mortality caused by PD during May–June (Figure 4). Mortality of the PIT‐tagged fish groups followed a similar pattern as the rest of the fish in the cage (Figure 3). The cumulative mortality of the PIT‐tag groups combined was only 4.2%, of which group AJm6 ended at 5.3% or approximately half that of the non‐marked fish in the cage also immunized with AJm6 (10.7%).

Of the 3,335 PIT‐tagged fish transferred to sea water, 2035 of these (61%) were sampled at the slaughterhouse with weights and fork lengths registered as shown in Table 2. The average weight of the Clm6 group (5.46 kg) was 0.5 kg heavier than the AJm6 control group (4.96 kg). The remaining groups all had lower average weight than the AJm6 group. Differences in sampling per cent of the different groups ranged from 58% for group A6 to 65% for group Clm6. Although none of the sampling percentages were significantly different from the control group AJm6, group Clm6 came closest with 5% higher rate (CI: ‐ 10.7–0.7, *p* = .09).

**TABLE 2:** Summary from harvest in the PIT‐tag study, including average harvest weight, confidence interval (CI) of harvest weight, length, sampling per cent, confidence interval of difference in sampling per cent from baseline and number of fish

Vaccine	Harvest weight (kg)	CI harvest weight	Length (cm)	Sampling per cent	CI diff from baseline	*N* (male/female)
AJm6	4.96	4.81, 5.10	75.7	60.0	Baseline	348 (187/161)
Clm6	5.46	5.33, 5.59	77.7	65.0	−10.7, 0.7	383 (193/190)
PDm6	4.79	4.63, 4.95	74.9	59.3	‐ 5.1, 6.7	336 (171/165)
Saline	4.81	4.64, 4.98	75.3	63.2	−9.2, 2,9	302 (145/156)^1^
PD7	4.95	4.81, 5.08	75.3	59.3	−6.4, 5.3	353 (181/172)
A6	4.84	4.70, 4.99	75.1	58.2	−4.1, 7.8	313 (165/147)^2^

Note

Sampling per cent is based on % of sea transferred fish in each group that were collected from the harvest line. ^1 and 2^ Sex missing from one observation.

In the regression analysis model, individual weight at vaccination, sex and runts had marked explanatory impact on harvest weight, increasing the adjusted R‐squared from 0.03 to 0.50. The estimates of the model using control group AJm6 (lacking a PD component) as the baseline are depicted in Table 3. Of all the groups, including the PD vaccines, only Clm6 had significantly greater harvest weight of 0.43 kg compared with control AJm6. Sex had large impact with the males estimated to be 1.09 kg heavier than the females, and each additional gram at vaccination provided an estimated 50 grams increase in harvest weight. Fish classified as runts were estimated to weigh 2.11 kg less compared with fish classified as normal.

**TABLE 3:** Results of OLS regression in the PIT study with harvest weight in kg as outcome variable

Explanatory variables	Estimate	CI	*p*‐value
Intercept	2.02	1.68, 2.36	<.01
Vaccine group (AJm6)			
Clm6	0.43	0.29, 0.57	<.01
PDm6	−0.01	−0.16, 0.13	.86
Saline	0.01	−0.15, 0.15	.93
PD7	−0.02	−0.17, 0.12	.75
A6	−0.10	−0.25, 0.05	.19
Sex (male)	1.09	1.00, 1.18	<.01
Weight vaccination (g)	0.05	0.04, 0.06	<.01
Runt	−2.11	−2.24, −0.1.98	<.01

Note

Control vaccine AJm6, as baseline among the vaccine groups, female as baseline in sex and not runt as baseline in runts. Total 2031 observation, four observations deleted due to missing values. Adjusted *R* ^2^ = .50.

The abdominal adhesion scores in the 5 PIT‐tagged fish groups immunized with oil‐adjuvanted vaccines (OAVs) were not significantly different, averaging from 1.9 in group Clm6 to 2.2 in group PDm6. While the abdominal adhesion scores in the groups immunized with OAVs were within the ranges as depicted in their respective SPCs, they measured significantly greater (*p* < .05) than those registered in the Saline group with an average score of 1.4.

### 4.3 Maxilla study

**FIGURE 5:**
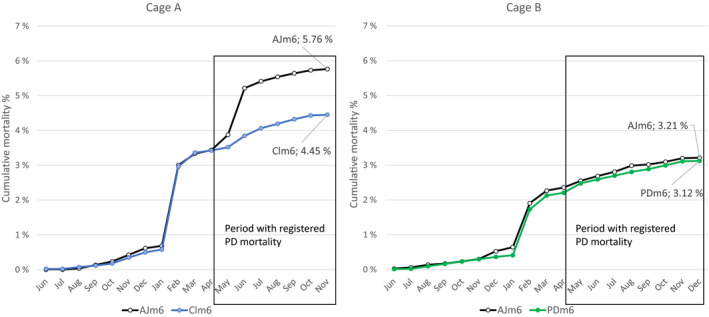
Cumulative mortality of the fish in cages A and B of the Maxilla study by group and month. Boxes indicate the period of mortality caused by PD in the two cages. Cumulative mortality in Clm6 group was significantly lower compared with the control group AJm6 (CI difference: −1.82%, −0.80%, *p* < .01)

Pancreas disease‐specific mortality was first registered in May in the Maxilla study cages A (Clm6 versus AJm6) and B (PDm5 versus AJm6). In cage A, the Clm6 group experienced significantly reduced cumulative mortality of 4.5% compared with control AJm6 with 5.8% (CI difference: −1.82%, −0.80% *p* < .01). The difference in mortality between the groups corresponds within the period with PD‐specific mortality. In cage B, no difference in mortality was registered between PDm6 and the control AJm6 (CI difference: −0.5%, 0.3% *p* = .70) (Figure 5). At harvest, the cumulative mortality had reached 9.1% in cage A and 6.2% for cage B of which 2.0% and 0.8% were caused by PD, respectively (Figure 3). The mortality of the maxilla clipped AJm6 control group in both cages was markedly lower than that of their unmarked counterparts: 5.8% versus 9.1% in cage A and 3.2% versus 6.1% in cage B (Figures 3 and 5).

**FIGURE 6:**
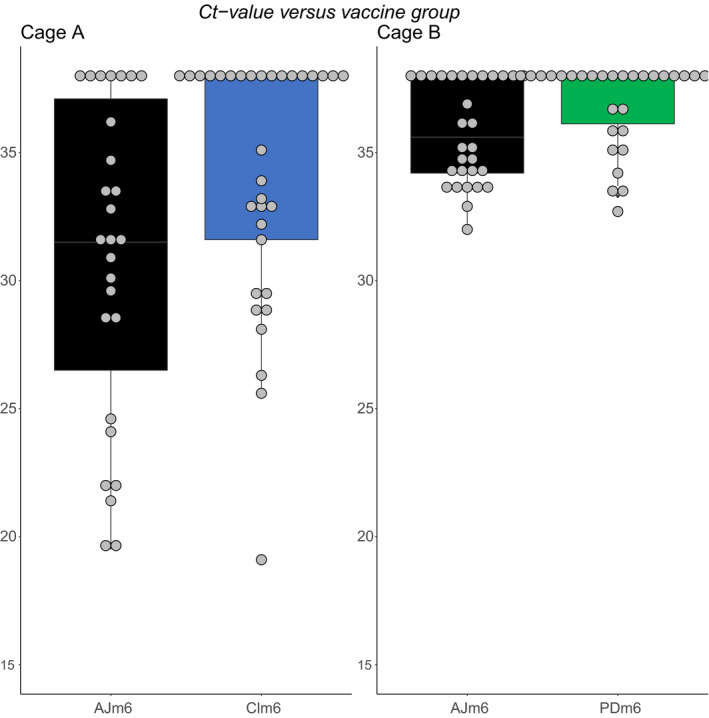
Box plots showing levels of SAV RNA as Ct values in fish from the Maxilla study sampled in June from in cage A, Clm6 (*n* = 33) versus AJm6 (*n* = 27), and in cage B, PDm6 (*n* = 30) versus AJm6 (*n* = 30). Results are presented as Ct values with a horizontal line within each box indicating the median, if less than 38. The box regions are limited by the 25th and 75th percentile, and whiskers mark the upper and lower adjacent value (1.5*inter‐quartile range). Both Clm6 (*p* = .03) and PDm6 (*p* = .03) difference significantly from AJm6

At the sampling on 24th June, that is during the PD outbreak, all the captured fish were counted to control for potential skewing in number of fish needed to collect targeted number of control‐ versus test group fish (left versus right maxilla clipped) in each of the cages. In Cage Maxilla A, a total of 313 fish were netted to get 33 marked fish from each of the groups. For Cage Maxilla B, 370 fish had to be netted to get 31 and 33 fish from each of the two groups. These results suggest non‐biased sampling between the test and control groups. The RT‐qPCR results demonstrated presence of SAV infections in both cages. Both PD‐vaccinated groups, Clm6 and PDm6, harboured significantly less SAV RNA levels (*p* = .03) in their hearts compared with their respective control counterparts in each of the cages (Figure 6). Abdominal adhesion evaluation of the same fish used for the RT‐qPCR analysis showed similar and normal scores averaging between 1.5 and 1.6. Examination of the vertebral columns of these fish using X‐ray imagery did not reveal any anomalies (data not shown).

In Cage A, the average harvest weight of the Clm6 group (5.58 kg) was 0.56 kg heavier than the AJm6 control group (5.02 kg). The regression analysis showed the Clm6 group to be significantly heavier (*p* < .01) than the control Ajm6 group with an estimated difference of 0.51 kg. In cage B, the average harvest weight of the PDm6 group including runts (5.02 kg) was 0.15 kg lighter than the AJm6 control group (5.22 kg). The regression analysis estimated the PDm6 group to be 0.12 kg lighter than the AJm6 controls (*p* = .10). In both cages, runts were significant as an explanatory variable estimated to –1.30 kg in cage A and –2.07 kg in cage B (Table 4).

**TABLE 4:** Result of OLS regression with harvest weight in kg as outcome variable in the Maxilla study

Cage	Explanatory variables	Estimate	CI	*p*‐value
Cage A	Intercept	5.23	5.12, 5.34	<.01
	Vaccine group (AJm6)			
	Clm6	0.51	0.36, 0.65	<.01
	Runt(no)	−1.30	−1.51, −1.09	<.01
Cage B	Intercept	5.48	5.38, 5.58	<.01
	Vaccine group (AJm6)			
	PDm6	−0.12	−0.26, 0.2	.10
	Runt	−2.07	−2.36, 1.78	<.01

Note

Control vaccine AJm6, not runt as baseline in runts. In Cage A, total of 885 (AJm6; 455 and Clm6; 430) observation and 1,000 (500 in each group) observations in Cage B. Adjusted *R* ^2^ = .19 in cage A and *R* ^2^ = .17 in cage B.

### 4.4 Cage study

The results from the Cage study with the fish harvested from July to November in 2019 are summarized in Table 5. Cumulative mortality until harvest in the 6 cages ranged from 13.2 in Cage 3%–6.0% in Cage 4 (both Clm6 groups). The fish in Cage 3 suffered higher transport‐related losses than the rest of the target population, counting for approximately ten per cent point of the cumulative mortality of 13.2% (Figure 3). Cumulative PD‐specific mortality in the 6 cages ranged from 0.2% to 3.9%. The Clm6 groups in Cages 3 and 4 experienced the lowest PD mortality of 0.2% and 0.9%, respectively. The Clm6 group in Cage 3 was harvested much earlier (14 June–3 July) than the remaining groups, and during a time when mortality caused by PD was still ongoing in the target population (Figure 3). This may, in part, explain why the lowest mortality caused by PD occurred in this cage (0.2%). Although the Clm6 group in Cage 4 was also harvested earlier than the remaining groups (12 August to 19 October), it was after the period of highest mortality caused by PD in the target population. Cumulative mortality until harvest caused by PD ranged from 2.5% to 3.9% in four cages containing the AJm6 and PDm6 groups. Harvest time point corresponds with the number of sea lice treatments for each cage, for the Clm6 group in Cages 3 (one treatment) and 4 (two treatments) compared with three to four treatments for the fish in the other 4 cages (Table 5).

**TABLE 5:** Summary of harvest data of the fish in the Cage study listed from earliest to last harvest dates

Vaccine group	Cage	Harvest period 2019	RGI	bFCR	Harvest weight (kg)	Normalized harvest weight (kg)	Number of treatments	Days of starving	Cum. Mortality	PD mortality	Superior
Clm6	3	14.06–03.07	96.9	1.10	3.69	5.78	1	27	13.2%	0.2%	96%
Clm6	4	12.08–19.10	96.3	1.18	5.11	5.60	2	27	6.0%	0.9%	98%
PDm6	1	24.10–04.11	94.7	1.26	5.30	5.12	4	31	9.5%	3.9%	98%
AJm6	5	14.06–06.11	95.0	1.22	4.99	5.17	3	38	10.0%	3.4%	98%
AJm6	6	01.11–18.11	91.8	1.27	5.18	4.52	4	42	8.6%	2.6%	98%
PDm6	2	24.10–24.11	94.9	1.24	5.69	5.15	3	37	7.5%	2.5%	98%

Note

The table shows last harvest date, growth (RGI, harvest weight(round) and normalized harvest weight), bFCR, number of treatments, number of days of starving, cumulative mortality, cumulative PD mortality and superior quality % at harvest

Performance of growth of the fish in the Cage study was sub‐optimal with RGI values ranging from 91.8 to 96.9, and normalized harvest weights averaging 5.23 kg (5.78–4.52), corresponding to more than a kg reduction in harvest weight compared with the expectation with an RGI 100 at 6.31 kg (Table 5). Based on normalized harvest weights, the fish in Cages 3 and 4 containing the Clm6 group ranked first and second, followed by Cages 5 (AJm6), 2 (PDm6), 1 (PDm6) and 6 (AJm6). A similar ranking between the vaccine groups was found for groups with the lowest bFCR. The harvest quality was registered during the slaughter process. While 96% of the fish in group Clm6 from Cage 3 were given the highest quality grade (“Superior”), 98% of the fish in the other 5 cages achieved the same quality status.

## 5 DISCUSSION

The efficacy of the PD vaccines was assessed by measuring the mortality and weight at harvest to determine the vaccines’ ability to prevent the growth impairment this disease causes (Kilburn et al., 2012; Pettersen et al., 2015; Røsæg et al., 2019; Taksdal et al., 2015; Thorarinsson et al., 2021). The target population used in these three studies, PIT‐tag, Maxilla and Cage study, suffered a PD outbreak due to a co‐infection with SAV 2 and SAV 3 with the clinical manifestation commencing approximately one year after sea transfer. Co‐infection of SAV subtypes at site level has recently been demonstrated in areas with overlapping geographical distribution of SAV subtypes (Gallagher et al., 2020). In Norway, co‐infections of the SAV 2 and SAV 3 were first reported in 2014 and later in 2019 and 2020 (Anonymous, 2020; Jansen et al., 2017). However, this is the first study that follows the development of prevalence of a site with co‐infection. SAV 2 dominated, measured as RNA prevalence in heart tissue suggesting that SAV 2 is more efficient in spreading the virus in the population (Figure 1). This is in accordance with an earlier finding where SAV 2 has been the dominating subtype in the fjord system of Romsdalen after the SAV2 introduction to the area in 2010 (Hjortaas et al., 2013, 2016). Further, both subtypes were detected in 10% of the samples with detection of SAV, indicating that co‐infection on the individual level is common in areas with both subtypes present.

The PIT‐tag study included three groups with PD vaccines (Clm6, PDm6 and PD7), two base vaccines lacking PD components as controls (AJm6 and A6) and a non‐vaccinated Saline group. The development of mortality in the PIT‐tagged fish followed the same pattern as the unmarked fish in the cage, with a marked peak in mortality related to the PD outbreak in June. The Clm6 group had the highest sampling per cent (65%) at the slaughterhouse, which was borderline non‐significant (*p* = .09) compared with the control (AJm6). The group with the second highest sampling per cent was the Saline group. The relatively low sampling per cent in the tagged fish compared with the registered mortality is most likely due to the manual collection of AFC fish from the harvest line, leading to a proportion of fish not detected. In the regression analysis controlling for weight at vaccination, sex and runts, only the Clm6 group differed significantly from the AJm6 control group with 0.43 kg (*p* < .01) increased harvest weight. No significant weight differences were found between the control vaccines, including the Saline group, and the other two PD vaccines.

Use of OAVs is reported to reduce growth of Atlantic salmon compared with their non‐vaccinated healthy counterparts by as much as 0.5 kg during a normal production cycle (Aunsmo, Larssen, et al., 2008; Midtlyng & Lillehaug, 1998). In contrast, and with exception of the Clm6 group, none of the groups immunized with OAVs demonstrated any growth penalty compared to the Saline group. The inherent nature of PD to negatively affect growth is the most likely explanation to this anomaly. A plausible explanation may further stem from multivalent OAVs inducing innate immune responses that cause temporary non‐specific protection against PD and as such leveraging the expected growth disadvantage compared with the Saline control group.

In the Maxilla study, the mortality development of the marked fish followed the same pattern as their unmarked counterparts in their respective cages. In Cage A of this study, the Clm6 group showed significantly less cumulative mortality (4.45%) (*p* < .01) compared with the AJm6 cohort counterpart (5.76%). This gap formed during the peak mortality period of the PD outbreak. The regression analysis demonstrated a similar significant effect on harvest weight between the Clm6 and AJm6 groups in the Maxilla study (+0.51 kg, *p* < .01), as in the PIT‐tag study (+0.43 kg, *p* < .01). In Cage B of the Maxilla study, no significant difference in mortality was registered between the PDm6 (3.12%) and the control AJm6 (3.21%). Harvest weight of the PDm6 group was further estimated to be 0.12 kg (*p* = .10) less than that of the AJm6 control group. The cause for lack of effect on mortality and inclination towards lower weight in the PDm6 test group cannot be determined. Plausible explanations include less impact of PD in this cage as suggested by the mortality levels during the peak outbreak and by the different infection dynamics as indicated by the RT‐qPCR results. The tendency towards lower weight of the PDm6 group may potentially be due to increased abdominal side effects caused by two OAVs with double the volume compared with the AJm6 controls. This potential increase in abdominal side effects was, however, not evident when measured 12 months post‐seawater transfer. Abdominal side effects are reported to peak around 6 months post‐seawater transfer and from then gradually decline in severity as the fish approach harvest weight (Mutoloki et al., 2004). Thus, the timing for the abdominal side effect assessment may not have been optimal. Growth may be a more sensitive parameter for the combined effect of the efficacy and the adverse effects than abdominal side effect assessment (Aunsmo, Larssen, et al., 2008).

Harvest weights registered in both the PIT‐tag and the Maxilla study revealed tendency towards a bimodal distribution at the left tale. This was evident in the residuals in the initial model, evaluated with a Q‐Q plot. To account for the tendency of a bimodal distribution K‐factor as a categorical variable runt (yes/no) was included to achieve normal distribution of the residuals. The inclusion of runts significantly improved the regression models and gave normal distributed residuals. The effect of runts in the models gave a reduction in the estimated effect of the vaccines on harvest weight. The causal direction of runts, vaccine and harvest weight is not necessary only one way. Runts is one of the classical manifestations of PD (McLoughlin & Graham, 2007), and if vaccine group affects the likelihood of runts, runts could be included as an outcome variable. This was not pursued further in this study, and the models with normal distributed residuals, highest adjusted *R* ^2^ and the most conservative vaccine effect were chosen as the final model. Although a reasonable hypothesis that an effective vaccine can affect the likelihood for runts after a PD outbreak, this should be pursued further in a specific designed study. Adverse vaccine side effects may also contribute to the development of runts but to address that question would have required a different experimental design than employed in these studies.

To evaluate abdominal adhesion scores, presence and relative magnitude of viral RNA, the fish sampled from the Maxilla study cages indicated no obvious skewed sampling numbers between the groups (Cage Maxilla A 33 versus 33 and Cage Maxilla B 31 versus 33). This kind of control of difference in distributions has low power with a test for equality of proportion. However, it can reveal if there are large differences in the likelihood of being sampled between the groups which can occur during a PD outbreak (Røsæg et al., 2018).

The viral analyses performed on the sampled fish in the Maxilla study after onset of clinical PD showed a lower viral load in the vaccine groups compared with their respective control group in the same cage. This is in accordance with previously published challenge studies for PD vaccines (Karlsen et al., 2012; Skjold et al., 2016; Thorarinsson et al., 2021). The difference was most apparent with a lower number of detections in the PD‐vaccinated groups. In fish with detections of SAV RNA, the Ct values were in the same range as the control group. The overall SAV RNA levels in the Maxilla study were lower in Cage B than in Cage A, indicating a higher infectious load in the latter. This may be due to asynchronous infection development, between the cages. This finding is supported by the dead and moribund fish in Cage B which when sampled in March revealed one of the highest prevalence and levels of SAV RNA (Ct range 20.5–31.5) (data not shown). In this cage, the measurement of viral loads in June, 3 months later, could have represented the tail end of the SAV infection (Andersen et al., 2007; Røsæg et al., 2019). The Ct values below 25 found in individual fish in cage A sampled at the same time in June was, however, more consistent with the viraemic phase in an ongoing PD infection.

The observed difference in cumulative mortalities between marked and unmarked fish vaccinated with the same vaccine combination, both in the PIT cage and in Cage A and B (Maxilla study), clearly indicates sampling bias. These differences were 5.8% versus 9.1%, 3.2% versus 6.1% and 5.3% versus 10.7% in Cages A, B and Cage PIT, respectively. This underlines the importance of similar marked test and control groups in field experiments. It also suggests that mortality results from field studies in large‐scale aquaculture cages where the design does not encompass control for sampling bias should be interpreted with care.

Immediately after the transportation of the target population to the SAV 3 endemic area, it showed reduced appetite and increased mortality. However, the fish health services, and the site manager did not consider PD as the main cause. The main clinical PD outbreak occurred from late May, seen as a second wave in prevalence of SAV RNA detections. This elongated period from detection to clinical outbreak is not uncommon (Jansen et al., 2010; Røsæg et al., 2019), it has been a previous observation that stressors such as livestock movement increase risk of clinical PD outbreaks in infected populations (Rodger & Mitchell, 2007). Although there was an increased mortality in connection to transportation, the overall mortality in the target population was deemed as moderate (8.9%) when compared to the median mortality in Norway for production cycles being harvested in 2019 being 13.5% (Sommerset et al., 2020).

In the descriptive Cage study, mortality caused by PD varied from 0.2% to 3.9% and cumulative mortality from 6.0% to 13.2%. The two cages vaccinated with Clm6 had the lowest PD mortality but ranked as top and bottom for accumulated mortality. Growth of the fish in the Cage study were sub‐optimal, with an averaged normalized harvest weight more than a kg below the expectations in the RGI model (5.23 kg versus expected 6.31 kg). This is likely partly due to PD, which is shown to cause marked growth reduction (Kilburn et al., 2012; Pettersen et al., 2015; Røsæg et al., 2019; Taksdal et al., 2015; Thorarinsson et al., 2021). However, the outcome of an infectious disease is a combination effect of the host, the environment and pathogen. Normalized harvest weight ranged from 4.52 to 5.78 in the Cage study and ranked the vaccines as in the PIT‐tag and the Maxilla studies (Table 5). With the highest normalized harvest weight in the Clm6‐vaccinated pens, increase in bFCR is also reported as an important manifestation of PD (Aunsmo et al., 2010; Pettersen et al., 2015; Røsæg et al., 2019). The Clm6 group cages provided lower bFCR than the remaining cages, thus further supporting the finding of improved protection against PD in this group. Numerous uncontrolled factors can influence the outcome when using limited number of whole cages in a descriptive study. The faster growth of the Clm6 group cages, which resulted in earlier harvesting, may have contributed positively to the mortality results of this group by reducing time exposed to SAV mortality, reduced PD‐related growth inhibition, less number of sea lice treatments and thus less number of days starved (Liu et al., 2014; Røsæg et al., 2019).

The data in this study are highly indicative of less disease and mortality and better weight gain for the fish that received the Clm6 vaccine. However, the RT‐qPCR (Figure 3) showed that both the Clm6‐ and PDm6‐vaccinated groups were infected with SAV, although with lower virus loads than the controls for both vaccines. Thus, the Clm6 vaccine did not block SAV infection, but it reduced infection levels and the severity of PD disease. The study was not designed to evaluate the effect or difference in effect of shedding of virus between PD vaccines. This should be investigated in designated studies.

## 6 CONCLUSION

In two controlled field studies, the efficacy of three commercially available PD vaccines was compared by measuring mortality and growth. For reference, a descriptive study employing six cages of fish was conducted in parallel. A natural, clinical PD outbreak was confirmed with presence of both SAV 2 and SAV 3. Only the group immunized with the Clm6 vaccine provided protection against mortality compared with the control group, significantly in one of the controlled field studies. Significant protection against PD‐induced loss of growth was similarly only found in the Clm6 group with increased harvest weight estimated at 0.43 and 0.51 kg compared with the control group of the two controlled field studies. The mortality levels caused by PD and growth performance in the descriptive Cage study aligned with the two controlled field studies.

## CONFLICT OF INTEREST

Magnus Vikan Røsæg is employed in SalMar (the owner of the fish included in the study). SalMar planed and conducted data registration in the study for internal use. After harvest and completed data registration, Elanco Animal Health, the manufacturer of the PD vaccine Clynav™, provided funding for the publication of the study. Ragnar Thorarinsson is employed by Elanco Animal Health.

## Supporting information

Supplementary Material

Supplementary Material

Supplementary Material

## Data Availability

The data that support the findings of this study are available from the corresponding author upon reasonable request.

## References

[jfd13505-bib-0001] Andersen, L. , Bratland, A. , Hodneland, K. , & Nylund, A. (2007). Tissue tropism of salmonid alphaviruses (subtypes SAV1 and SAV3) in experimentally challenged Atlantic salmon (*Salmo salar* L.). Archives of Virology, 152, 1871–1883. 10.1007/s00705-007-1006-1 17578649

[jfd13505-bib-0002] Anonymous . (2017). Forskrift om tiltak for å forebygge, begrense og bekjempe pankreassykdom (PD) hos akvakulturdyr. https://lovdata.no/dokument/SF/forskrift/2017‐08‐29‐1318?q=pd

[jfd13505-bib-0003] Anonymous . (2020). Pankreassykdom(PD)‐ utbrudd og statsikk. Norwegian Veterinary Institute., https://www.vetinst.no/dyr/oppdrettsfisk/pankreassykdom‐pd‐utbrudd‐og‐statistikk

[jfd13505-bib-0004] Aunsmo, A. , Bruheim, T. , Sandberg, M. , Skjerve, E. , Romstad, S. , & Larssen, R. B. (2008). Methods for investigating patterns of mortality and quantifying cause‐specific mortality in sea‐farmed Atlantic salmon *Salmo salar* . Diseases of Aquatic Organisms, 81, 99–107. 10.3354/dao01954 18924374

[jfd13505-bib-0005] Aunsmo, A. , Larssen, R. B. , Valle, P. S. , Sandberg, M. , Evensen, Ø. , Midtlyng, P. J. , Østvik, A. , & Skjerve, E. (2008). Improved field trial methodology for quantifying vaccination side‐effects in farmed Atlantic salmon (*Salmo salar* L.). Aquaculture, 284, 19–24. 10.1016/j.aquaculture.2008.07.028

[jfd13505-bib-0006] Aunsmo, A. , Valle, P. S. , Sandberg, M. , Midtlyng, P. J. , & Bruheim, T. (2010). Stochastic modelling of direct costs of pancreas disease (PD) in Norwegian farmed Atlantic salmon (*Salmo salar* L.). Preventive Veterinary Medicine, 93, 233–241. 10.1016/j.prevetmed.2009.10.001 19931201

[jfd13505-bib-0007] Bang Jensen, B. , Kristoffersen, A. B. , Myr, C. , & Brun, E. (2012). Cohort study of effect of vaccination on pancreas disease in Norwegian salmon aquaculture. Diseases of Aquatic Organisms, 102, 23–31. 10.3354/dao02529 23209075

[jfd13505-bib-0008] Chang, C. J. , Gu, J. , & Robertsen, B. (2017). Protective effect and antibody response of DNA vaccine against salmonid alphavirus 3 (SAV3) in Atlantic salmon. Journal of Fish Diseases, 40, 1–7. 10.1111/jfd.12644 28493514

[jfd13505-bib-0009] Fringuelli, E. , Rowley, H. M. , Wilson, J. C. , Hunter, R. , Rodger, H. , & Graham, D. A. (2008). Phylogenetic analyses and molecular epidemiology of European salmonid alphaviruses (SAV) based on partial E2 and nsP3 gene nucleotide sequences. Journal of Fish Diseases, 31, 811–823. 10.1111/j.1365-2761.2008.00944.x 18681902

[jfd13505-bib-0010] Gallagher, M. D. , Matejusova, I. , Ruane, N. M. , & Macqueen, D. J. (2020). Genome‐wide target enriched viral sequencing reveals extensive ‘hidden’ salmonid alphavirus diversity in farmed and wild fish populations. Aquaculture, 522, 10.1016/j.aquaculture.2020.735117

[jfd13505-bib-0011] Graham, D. A. , Frost, P. , McLaughlin, K. , Rowley, H. M. , Gabestad, I. , Gordon, A. , & McLoughlin, M. F. (2011). A comparative study of marine salmonid alphavirus subtypes 1–6 using an experimental cohabitation challenge model. Journal of Fish Diseases, 34, 273–286. 10.1111/j.1365-2761.2010.01234.x 21294751

[jfd13505-bib-0012] Graham, D. A. , Rowley, H. R. , & Frost, P. (2014). Cross‐neutralization studies with salmonid alphavirus subtype 1–6 strains: Results with sera from experimental studies and natural infections. Journal of Fish Diseases, 37, 683–691. 10.1111/jfd.12167 23957811

[jfd13505-bib-0013] Hikke, M. C. , Braaen, S. , Villoing, S. , Hodneland, K. , Geertsema, C. , Verhagen, L. , Frost, P. , Vlak, J. M. , Rimstad, E. , & Pijlman, G. P. (2014). Salmonid alphavirus glycoprotein E2 requires low temperature and E1 for virion formation and induction of protective immunity. Vaccine, 32, 6206–6212. 10.1016/j.vaccine.2014.09.026 25269093

[jfd13505-bib-0014] Hjortaas, M. J. , Bang Jensen, B. , Taksdal, T. , Olsen, A. B. , Lillehaug, A. , Trettenes, E. , & Sindre, H. (2016). Genetic characterization of salmonid alphavirus in Norway. Journal of Fish Diseases, 39, 249–257. 10.1111/jfd.12353 25683753

[jfd13505-bib-0015] Hjortaas, M. J. , Skjelstad, H. R. , Taksdal, T. , Olsen, A. B. , Johansen, R. , Bang‐Jensen, B. , Ørpetveit, I. , Sindre, H. , Orpetveit, I. , & Sindre, H. (2013). The first detections of subtype 2‐related salmonid alphavirus (SAV2) in Atlantic salmon, *Salmo salar* L., in Norway. Journal of Fish Diseases, 36, 71–74. 10.1111/j.1365-2761.2012.01445.x 22943794

[jfd13505-bib-0016] Jansen, M. D. , Bang Jensen, B. , McLoughlin, M. F. , Rodger, H. D. , Taksdal, T. , Sindre, H. , Graham, D. A. , & Lillehaug, A. (2017). The epidemiology of pancreas disease in salmonid aquaculture: A summary of the current state of knowledge. Journal of Fish Diseases, 40, 141–155. 10.1111/jfd.12478 27136332

[jfd13505-bib-0017] Jansen, M. D. , Jensen, B. B. , & Brun, E. (2015). Clinical manifestations of pancreas disease outbreaks in Norwegian marine salmon farming ‐ Variations due to salmonid alphavirus subtype. Journal of Fish Diseases, 38, 343–353. 10.1111/jfd.12238 24661057

[jfd13505-bib-0018] Jansen, M. D. , Wasmuth, M. A. , Olsen, A. B. , Gjerset, B. , Modahl, I. , Breck, O. , Haldorsen, R. N. , Hjelmeland, R. , & Taksdal, T. (2010). Pancreas disease (PD) in sea‐reared Atlantic salmon, *Salmo salar* L., in Norway; a prospective, longitudinal study of disease development and agreement between diagnostic test results. Journal of Fish Diseases, 33, 723–736. 10.1111/j.1365-2761.2010.01176.x 20609035

[jfd13505-bib-0019] Johansen, L.‐H.‐H. , Thim, H. L. , Jørgensen, S. M. , Afanasyev, S. , Strandskog, G. , Taksdal, T. , Fremmerlid, K. , McLoughlin, M. , Jørgensen, J. B. , & Krasnov, A. (2015). Comparison of transcriptomic responses to pancreas disease (PD) and heart and skeletal muscle inflammation (HSMI) in heart of Atlantic salmon (*Salmo salar* L). Fish & Shellfish Immunology, 46, 612–623. 10.1016/j.fsi.2015.07.023 26232631

[jfd13505-bib-0020] Karlsen, M. , Tingbø, T. , Solbakk, I.‐T. , Evensen, O. , Furevik, A. , & Aas‐Eng, A. (2012). Efficacy and safety of an inactivated vaccine against Salmonid alphavirus (family Togaviridae). Vaccine, 30, 5688–5694. 10.1016/j.vaccine.2012.05.069 22691434

[jfd13505-bib-0021] Kilburn, R. , Murray, A. G. , Hall, M. , Bruno, D. W. , Cockerill, D. , & Raynard, R. S. (2012). Analysis of a company’s production data to describe the epidemiology and persistence of pancreas disease in Atlantic salmon (*Salmo salar* L.) farms off Western Scotland. Aquaculture, 368, 89–94. 10.1016/j.aquaculture.2012.09.004

[jfd13505-bib-0022] Lewisch, E. , Frank, T. , Soliman, H. , Schachner, O. , Friedl, A. , & El‐Matbouli, M. (2018). First confirmation of salmonid alphavirus infection in Arctic char *Salvelinus alpinus* and in Austria. Diseases of Aquatic Organisms, 130, 71–76. 10.3354/dao03265 30154274

[jfd13505-bib-0023] Liu, Y. , & Bjelland, H. V. (2014). Estimating costs of sea lice control strategy in Norway. Preventive Veterinary Medicine., 117, 469–477. 10.1016/j.prevetmed.2014.08.018 25443395

[jfd13505-bib-0024] López‐Dóriga, M. V. , Smail, D. A. , Smith, R. J. , Doménech, A. , Castric, J. , Smith, P. D. , & Ellis, A. (2001). Isolation of salmon pancreas disease virus (SPDV) in cell culture and its ability to protect against infection by the “wild‐type” agent. Fish & Shellfish Immunology, 11, 505–522. 10.1006/fsim.2000.0330 11556480

[jfd13505-bib-0025] McLoughlin, M. F. , & Graham, D. A. (2007). Alphavirus infections in salmonids ‐ A review. Journal of Fish Diseases, 30, 511–531. 10.1111/j.1365-2761.2007.00848.x 17718707

[jfd13505-bib-0026] Midtlyng, P. J. , & Lillehaug, A. (1998). Growth of Atlantic salmon *Salmo salar* after intraperitoneal administration of vaccines containing adjuvants. Disease of Aquatic Organisms, 32, 91–97. 10.3354/dao032091 9696628

[jfd13505-bib-0027] Midtlyng, P. J. , Reitan, L. J. , & Speilberg, L. (1996). Experimental studies on the efficacy and side‐effects of intraperitoneal vaccination of Atlantic salmon (*Salmo salar* L.) against furunculosis. Fish and Shellfish Immunology, 6, 335–350. 10.1006/fsim.1996.0034

[jfd13505-bib-0028] Munro, A. L. S. , Ellis, A. E. , McVicar, A. H. , & Needham, E. A. (1984). An exocrine disease of farmed Atlantic salmon in Scotland. Helgoländer Meeresunters, 37, 571–586.

[jfd13505-bib-0029] Mutoloki, S. , Alexandersen, S. , & Evensen, Ø. (2004). Sequential study of antigen persistence and concomitant inflammatory reactions relative to side‐effects and growth of Atlantic salmon (*Salmo salar* L.) following intraperitoneal injection with oil‐adjuvanted vaccines. Fish and Shellfish Immunology, 16, 633–644. 10.1016/j.fsi.2003.10.002 15110337

[jfd13505-bib-0030] Pettersen, J. M. , Rich, K. M. , Bang Jensen, B. , & Aunsmo, A. (2015). The economic benefits of disease triggered early harvest: A case study of pancreas disease in farmed Atlantic salmon from Norway. Preventive Veterinary Medicine, 121, 314–324. 10.1016/j.prevetmed.2015.08.003 26297077

[jfd13505-bib-0031] Rodger, H. , & Mitchell, S. (2007). Epidemiological observations of pancreas disease of farmed Atlantic salmon, *Salmo salar* L., in Ireland. Journal of Fish Diseases, 30, 157–167. 10.1111/j.1365-2761.2007.00799.x 17352791

[jfd13505-bib-0032] Røsæg, M. V. , Garseth, Å. H. , Brynildsrud, O. B. , & Jansen, M. D. (2019). Pancreas disease caused by Salmonid alphavirus subtype 2 reduces growth and feed conversion in farmed Atlantic salmon. Preventive Veterinary Medicine, 169, 104699. 10.1016/j.prevetmed.2019.104699 31311646

[jfd13505-bib-0033] Røsæg, M. V. , Rimstad, E. , Guttvik, A. , Skjelstad, B. , Bendiksen, E. Å. , & Garseth, Å. H. (2018). Effect of pancreas disease caused by SAV 2 on protein and fat digestion in Atlantic salmon. Journal of Fish Diseases, 42(1), 97–108. 10.1111/jfd.12914 30370677

[jfd13505-bib-0034] RStudio, T . (2020). RStudio: Integrated development for R. (PBC). Rstudio. http://www.rstudio.com/

[jfd13505-bib-0035] Skjold, P. , Sommerset, I. , Frost, P. , & Villoing, S. (2016). Vaccination against pancreas disease in Atlantic salmon, *Salmo salar* L., reduces shedding of salmonid alphavirus. Veterinary Research, 47, 10–15. 10.1186/s13567-016-0362-9 27496170 PMC4975881

[jfd13505-bib-0036] Sommerset, I. , Walde, C. S. , Bang Jensen, B. , Bornø, G. , Haukaas, A. , & Brun, E. (2020). The health situation in Norwegian aquaculture 2019. Norwegian Veterinary Institute. https://www.vetinst.no/rapporter‐og‐publikasjoner/rapporter/2020/fish‐health‐report‐2019

[jfd13505-bib-0037] Taksdal, T. , Bang Jensen, B. , Bockerman, I. , McLoughlin, M. F. , Hjortaas, M. J. , Ramstad, A. , & Sindre, H. (2015). Mortality and weight loss of Atlantic salmon, *Salmon salar* L., experimentally infected with salmonid alphavirus subtype 2 and subtype 3 isolates from Norway. Journal of Fish Diseases, 38, 1047–1061. 10.1111/jfd.12312 25322679

[jfd13505-bib-0038] Thim, H. L. , Iliev, D. B. , Christie, K. E. , Villoing, S. , McLoughlin, M. F. , Strandskog, G. , & Jørgensen, J. B. (2012). Immunoprotective activity of a Salmonid Alphavirus Vaccine: Comparison of the immune responses induced by inactivated whole virus antigen formulations based on CpG class B oligonucleotides and poly I: C alone or combined with an oil adjuvant. Vaccine, 30, 4828–4834. 10.1016/j.vaccine.2012.05.010 22634299

[jfd13505-bib-0039] Thorarinsson, R. , Wolf, J. C. , Inami, M. , Phillips, L. , Jones, G. , Macdonald, A. M. , Rodriguez, J. F. , Sindre, H. , Skjerve, E. , Rimstad, E. , & Evensen, Ø. (2021). Effect of a novel DNA vaccine against pancreas disease caused by salmonid alphavirus subtype 3 in Atlantic salmon (*Salmon salar*). Fish & Shellfish Immunology, 108, 116–126. 10.1016/j.fsi.2020.12.002 33285168

[jfd13505-bib-0040] Tighe, A. A. J. , Gallagher, M. M. D. , Carlsson, J. , Matejusova, I. , Swords, F. , Macqueen, D. J. D. , & Ruane, N. N. M. (2020). Nanopore whole genome sequencing and partitioned phylogenetic analysis supports a new salmonid alphavirus genotype (SAV7). Diseases of Aquatic Organisms, 142, 203–211. 10.3354/dao03546 33331288

[jfd13505-bib-0041] Villoing, S. , Bearzotti, M. , Chilmonczyk, S. , Castric, J. , & Bremont, M. (2000). Rainbow Trout Sleeping Disease Virus Is an Atypical Alphavirus. Journal of Virology, 74, 173–183. 10.1128/JVI.74.1.173-183.2000 10590104 PMC111526

[jfd13505-bib-0042] Xu, C. , Mutoloki, S. , Evensen, Ø. , & Evensen, O. (2012). Superior protection conferred by inactivated whole virus vaccine over subunit and DNA vaccines against salmonid alphavirus infection in Atlantic salmon (*Salmo salar* L.). Vaccine, 30, 3918–3928. 10.1016/j.vaccine.2012.03.081 22504037

