# Supplementary figures and images for: Effect of vaccines against pancreas disease in farmed Atlantic salmon
Source: J Fish Dis. 2021 Aug 17;44(12):1911–24. doi: 10.1111/jfd.13505 (PMC9291808; doi:10.1111/jfd.13505)

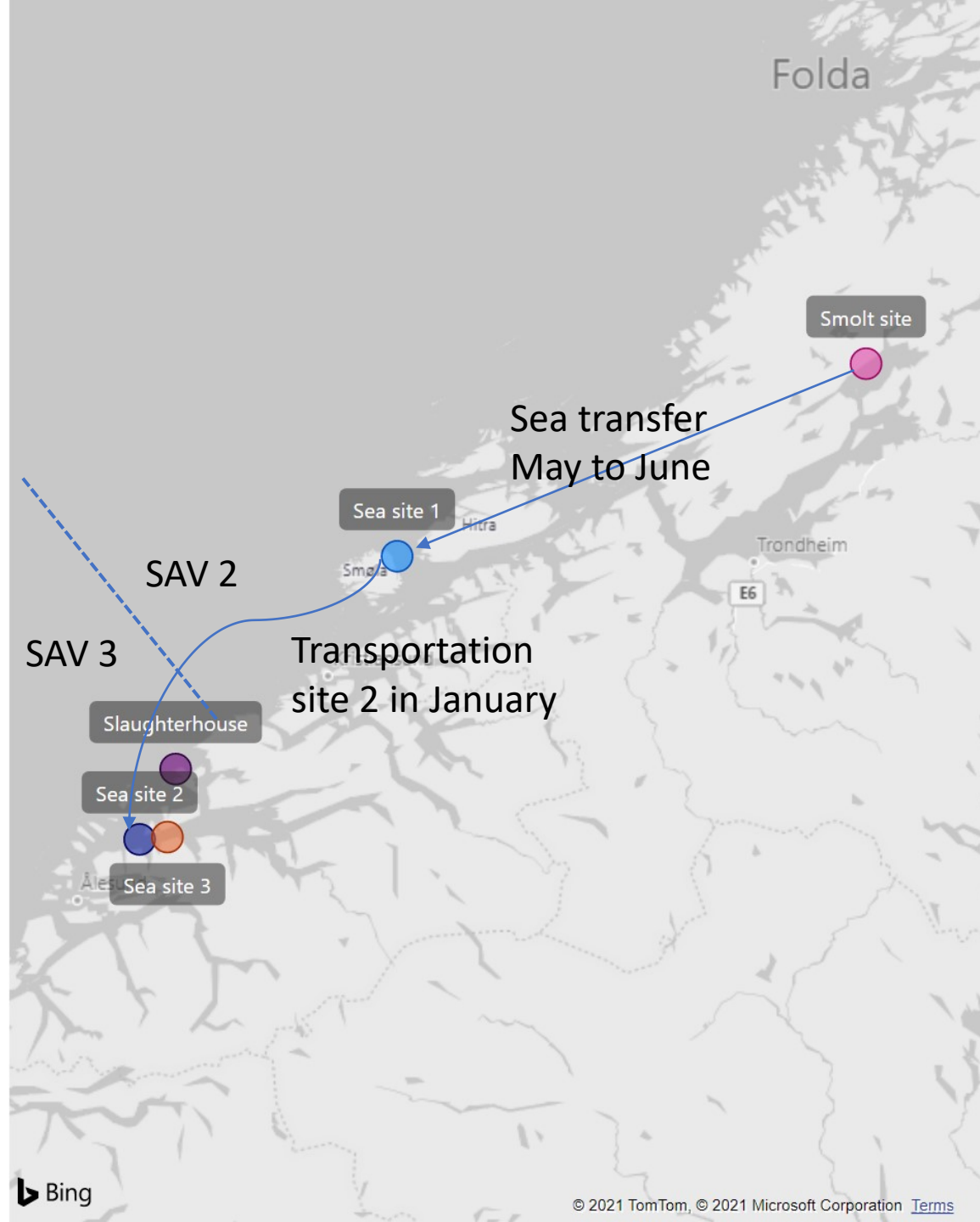

Supplement: Supplementary file 1 — Supplementary Material [file JFD-44-1911-s003.pdf]
