# Supplementary material for: Effect of vaccines against pancreas disease in farmed Atlantic salmon
Source: J Fish Dis. 2021 Aug 17;44(12):1911–24. doi: 10.1111/jfd.13505 (PMC9291808; doi:10.1111/jfd.13505)
Supplement: Supplementary file 2 — Supplementary Material [file JFD-44-1911-s001.pdf]

Pens and site / Physical unit

| Year | | 2018 | | | | | | | | | | | | | | | | | | | | | | | | | | | | | | | | | | | |
|---|---|---|---|---|---|---|---|---|---|---|---|---|---|---|---|---|---|---|---|---|---|---|---|---|---|---|---|---|---|---|---|---|---|---|---|---|---|
| Week | | 18 | 19 | 20 | 21 | 22 | 23 | 24 | 25 | 26 | 27 | 28 | 29 | 30 | 31 | 32 | 33 | 34 | 35 | 36 | 37 | 38 | 39 | 40 | 41 | 42 | 43 | 44 | 45 | 46 | 47 | 48 | 49 | 50 | 51 | 52 | 53 |
| 1 | Reiråklakken | | | 1 | 1 | 1 | 1 | 1 | 1 | 1 | 1 | 1 | 1 | 1 | 1 | 1 | 1 | 1 | 1 | 1 | 1 | 1 | 1 | 1 | 1 | 1 | 1 | 1 | 1 | 1 | 1 | 1 | 1 | 1 | 1 | 1 | 1 |
| | Terningen | | | | | | | | | | | | | | | | | | | | | | | | | | | | | | | | | | | | |
| 2 | Dryna | | | | | | | | | | | | | | | | | | | | | | | | | | | | | | | | | | | | |
| | Reiråklakken | | | 2 | 2 | 2 | 2 | 2 | 2 | 2 | 2 | 2 | 2 | 2 | 2 | 2 | 2 | 2 | 2 | 2 | 2 | 2 | 2 | 2 | 2 | 2 | 2 | 2 | 2 | 2 | 2 | 2 | 2 | 2 | 2 | 2 | 2 |
| 3 | Dryna | | | | | | | | | | | | | | | | | | | | | | | | | | | | | | | | | | | | |
| | Reiråklakken | 5 | 5 | 5 | 5 | 5 | 5 | 5 | 5 | 5 | 5 | 5 | 5 | 5 | 5 | 5 | 5 | 5 | 5 | 5 | 5 | 5 | 5 | 5 | 5 | 5 | 5 | 5 | 5 | 5 | 5 | 5 | 5 | 5 | 5 | 5 | 5 |
| 4 | Reiråklakken | 6 | 6 | 6 | 6 | 6 | 6 | 6 | 6 | 6 | 6 | 6 | 6 | 6 | 6 | 6 | 6 | 6 | 6 | 6 | 6 | 6 | 6 | 6 | 6 | 6 | 6 | 6 | 6 | 6 | 6 | 6 | 6 | 6 | 6 | 6 | 6 |
| | Terningen | | | | | | | | | | | | | | | | | | | | | | | | | | | | | | | | | | | | |
| 5 | Myrane | | | | | | | | | | | | | | | | | | | | | | | | | | | | | | | | | | | | |
| | Reiråklakken | 3 | 3 | 3 | 3 | 3 | 3 | 3 | 3 | 3 | 3 | 3 | 3 | 3 | 3 | 3 | 3 | 3 | 3 | 3 | 3 | 3 | 3 | 3 | 3 | 3 | 3 | 3 | 3 | 3 | 3 | 3 | 3 | 3 | 3 | 3 | 3 |
| | Terningen | | | | | | | | | | | | | | | | | | | | | | | | | | | | | | | | | | | | |
| 6 | Dryna | | | | | | | | | | | | | | | | | | | | | | | | | | | | | | | | | | | | |
| | Myrane | | | | | | | | | | | | | | | | | | | | | | | | | | | | | | | | | | | | |
| | Reiråklakken | 4 | 4 | 4 | 4 | 4 | 4 | 4 | 4 | 4 | 4 | 4 | 4 | 4 | 4 | 4 | 4 | 4 | 4 | 4 | 4 | 4 | 4 | 4 | 4 | 4 | 4 | 4 | 4 | 4 | 4 | 4 | 4 | 4 | 4 | 4 | 4 |
| A | Dryna | | | | | | | | | | | | | | | | | | | | | | | | | | | | | | | | | | | | |
| | Fuglåsen | | | | | | | | 13 | 13 | 13 | 13 | 13 | 13 | 13 | 13 | 13 | 13 | 13 | 13 | 13 | 13 | 13 | 13 | 13 | 13 | 13 | 13 | 13 | 13 | 13 | 13 | 13 | 13 | 13 | 13 | 13 |
| B | Fuglåsen | | | | | | | | 14 | 14 | 14 | 14 | 14 | 14 | 14 | 14 | 14 | 14 | 14 | 14 | 14 | 14 | 14 | 14 | 14 | 14 | 14 | 14 | 14 | 14 | 14 | 14 | 14 | 14 | 14 | 14 | 14 |
| | Terningen | | | | | | | | | | | | | | | | | | | | | | | | | | | | | | | | | | | | |
| PIT | Dryna | | | | | | | | | | | | | | | | | | | | | | | | | | | | | | | | | | | | |
| | Fuglåsen | | | | | | | | 12 | 12 | 12 | 12 | 12 | 12 | 12 | 12 | 12 | 12 | 12 | 12 | 12 | 12 | 12 | 12 | 12 | 12 | 12 | 12 | 12 | 12 | 12 | 12 | 12 | 12 | 12 | 12 | 12 |

| Year | | 2019 | | | | | | | | | | | | | | | | | | | | | | | | | | | | | | | | | | | | | | | | | | | | | | | | | |
|---|---|---|---|---|---|---|---|---|---|---|---|---|---|---|---|---|---|---|---|---|---|---|---|---|---|---|---|---|---|---|---|---|---|---|---|---|---|---|---|---|---|---|---|---|---|---|---|---|---|---|---|---|
| Week | | 1 | 2 | 3 | 4 | 5 | 6 | 7 | 8 | 9 | 10 | 11 | 12 | 13 | 14 | 15 | 16 | 17 | 18 | 19 | 20 | 21 | 22 | 23 | 24 | 25 | 26 | 27 | 28 | 29 | 30 | 31 | 32 | 33 | 34 | 35 | 36 | 37 | 38 | 39 | 40 | 41 | 42 | 43 | 44 | 45 | 46 | 47 | 48 | 49 | 50 | 51 |
| 1 | Reiråklakken | 1 | 1 | 1 | 1 | 1 | | | | | | | | | | | | | | | | | | | | | | | | | | | | | | | | | | | | | | | | | | | | | | |
| | Terningen | | | | | 12 | 12 | 12 | 12 | 12 | 12 | 12 | 12 | 12 | 12 | 12 | 12 | 12 | 12 | 12 | 12 | 12 | 12 | 12 | 12 | 22 | 22 | 22 | 22 | 22 | 21 | 21 | 21 | 21 | 21 | 22 | 22 | 22 | 22 | 22 | 21 | 21 | 21 | 21 | 21 | 21 | | | | | | |
| 2 | Dryna | | | | | 41 | 41 | 41 | 41 | 41 | 41 | 41 | 41 | 41 | 41 | 41 | 41 | 41 | 41 | 41 | 41 | 41 | 41 | 41 | 41 | 51 | 51 | 51 | 51 | 51 | 41 | 41 | 41 | 41 | 41 | 41 | 41 | 41 | 41 | 41 | 41 | 31 | 31 | 31 | 31 | 31 | 31 | 31 | | | | |
| | Reiråklakken | 2 | 2 | 2 | 2 | 2 | | | | | | | | | | | | | | | | | | | | | | | | | | | | | | | | | | | | | | | | | | | | | | |
| 3 | Dryna | | | | 32 | 32 | 32 | 32 | 32 | 32 | 32 | 32 | 32 | 32 | 32 | 32 | 32 | 32 | 32 | 32 | 32 | 32 | 33 | 33 | 33 | 33 | 33 | | | | | | | | | | | | | | | | | | | | | | | | | |
| | Reiråklakken | 5 | 5 | 5 | 5 | | | | | | | | | | | | | | | | | | | | | | | | | | | | | | | | | | | | | | | | | | | | | | | |
| 4 | Reiråklakken | 6 | 6 | 6 | 6 | | | | | | | | | | | | | | | | | | | | | | | | | | | | | | | | | | | | | | | | | | | | | | | |
| | Terningen | | | | 11 | 11 | 11 | 11 | 11 | 11 | 11 | 11 | 11 | 11 | 11 | 11 | 11 | 11 | 11 | 11 | 11 | 11 | 11 | 11 | 11 | 12 | 12 | 12 | 12 | 12 | 12 | 12 | 12 | 12 | 12 | 12 | 12 | 12 | 12 | 12 | 11 | 11 | 11 | | | | | | | | | |
| 5 | Myrane | | | | | | | | | | | | | | | | | | | | | | | | | | | | | | | | | | | | | | | 13 | 13 | 14 | 14 | 14 | 14 | 14 | | | | | | |
| | Reiråklakken | 3 | 3 | 3 | 3 | 3 | | | | | | | | | | | | | | | | | | | | | | | | | | | | | | | | | | | | | | | | | | | | | | |
| | Terningen | | | | | 21 | 21 | 21 | 21 | 21 | 21 | 21 | 21 | 21 | 21 | 21 | 21 | 21 | 21 | 21 | 21 | 21 | 21 | 21 | 11 | 11 | 11 | 11 | 11 | 11 | 11 | 11 | 11 | 11 | 11 | 21 | 21 | 21 | 21 | 21 | | | | | | | | | | | | |
| 6 | Dryna | | | | 33 | 33 | 33 | 33 | 33 | 33 | 33 | 33 | 33 | 33 | 33 | 33 | 33 | 33 | 33 | 33 | 33 | 33 | 33 | 34 | 34 | 34 | 34 | 34 | 34 | 34 | 33 | 33 | 33 | 33 | 33 | 34 | 34 | 34 | 34 | 34 | | | | | | | | | | | | |
| | Myrane | | | | | | | | | | | | | | | | | | | | | | | | | | | | | | | | | | | | | | | | 14 | 15 | 15 | 15 | 15 | 15 | 15 | 15 | | | | |
| | Reiråklakken | 4 | 4 | 4 | 4 | | | | | | | | | | | | | | | | | | | | | | | | | | | | | | | | | | | | | | | | | | | | | | | |
| A | Dryna | | | | | 51 | 51 | 51 | 51 | 51 | 51 | 51 | 51 | 51 | 51 | 51 | 51 | 51 | 51 | 51 | 51 | 51 | 51 | 51 | 52 | 52 | 52 | 52 | 52 | 52 | 51 | 51 | 51 | 51 | 51 | 51 | 51 | 51 | 51 | 51 | 51 | 41 | 41 | 41 | 41 | 41 | 41 | 41 | 41 | | | |
| | Fuglåsen | 13 | 13 | 13 | 13 | 13 | | | | | | | | | | | | | | | | | | | | | | | | | | | | | | | | | | | | | | | | | | | | | | |
| B | Fuglåsen | 14 | 14 | 14 | 14 | 14 | | | | | | | | | | | | | | | | | | | | | | | | | | | | | | | | | | | | | | | | | | | | | | |
| | Terningen | | | | | 22 | 22 | 22 | 22 | 22 | 22 | 22 | 22 | 22 | 22 | 22 | 22 | 22 | 22 | 22 | 22 | 22 | 22 | 23 | 23 | 23 | 23 | 23 | 23 | 23 | 22 | 22 | 22 | 22 | 22 | 23 | 23 | 23 | 23 | 23 | 22 | 22 | 22 | 22 | 22 | 22 | 22 | 22 | 23 | 23 | 23 | 23 |
| PIT | Dryna | | | | | 52 | 52 | 52 | 52 | 52 | 52 | 52 | 52 | 52 | 52 | 52 | 52 | 52 | 52 | 52 | 52 | 52 | 52 | 52 | 53 | 53 | 53 | 53 | 53 | 53 | 53 | 52 | 52 | 52 | 52 | 53 | 53 | 53 | 53 | 52 | 52 | 52 | 52 | 52 | 52 | 52 | 52 | 51 | 51 | 51 | | |
| | Fuglåsen | 12 | 12 | 12 | 12 | 12 | | | | | | | | | | | | | | | | | | | | | | | | | | | | | | | | | | | | | | | | | | | | | | |

Schematic presentation of pens, sites and physical units in the target population by week number.
