# Supplementary material for: Effect of vaccines against pancreas disease in farmed Atlantic salmon
Source: J Fish Dis. 2021 Aug 17;44(12):1911–24. doi: 10.1111/jfd.13505 (PMC9291808; doi:10.1111/jfd.13505)
Supplement: Supplementary file 3 — Supplementary Material [file JFD-44-1911-s002.pdf]

AQUASIM™

SGR % pr day Atlantic Salmon (*Salmon Salar*)

Temperature

Weight	2	3	4	5	6	7	8	9	10	11	12	13	14	15	16	17	18	FCR	acc FCR
50	0.46	0.69	0.91	1.11	1.30	1.46	1.62	1.76	1.90	2.02	2.12	2.20	2.23	2.20	2.10	1.91	1.61	0.80	0.80
60	0.46	0.70	0.92	1.12	1.30	1.46	1.62	1.76	1.89	2.01	2.11	2.18	2.20	2.17	2.07	1.88	1.59	0.80	0.80
70	0.47	0.70	0.92	1.12	1.29	1.46	1.61	1.75	1.88	1.99	2.08	2.15	2.17	2.14	2.04	1.85	1.56	0.80	0.80
80	0.46	0.70	0.91	1.11	1.29	1.45	1.60	1.73	1.86	1.97	2.06	2.12	2.14	2.11	2.00	1.82	1.54	0.80	0.80
90	0.46	0.69	0.90	1.10	1.28	1.43	1.58	1.71	1.84	1.94	2.03	2.09	2.11	2.07	1.97	1.78	1.51	0.80	0.80
100	0.46	0.68	0.90	1.09	1.26	1.42	1.56	1.70	1.82	1.92	2.01	2.06	2.08	2.04	1.94	1.75	1.48	0.81	0.80
110	0.45	0.68	0.89	1.08	1.25	1.41	1.55	1.68	1.79	1.90	1.98	2.03	2.05	2.01	1.91	1.73	1.46	0.81	0.80
120	0.45	0.67	0.88	1.07	1.24	1.39	1.53	1.66	1.77	1.87	1.95	2.00	2.02	1.98	1.88	1.70	1.43	0.81	0.80
130	0.44	0.66	0.87	1.05	1.22	1.37	1.51	1.64	1.75	1.85	1.93	1.98	1.99	1.95	1.85	1.67	1.41	0.81	0.80
140	0.44	0.65	0.86	1.04	1.21	1.36	1.50	1.62	1.73	1.83	1.90	1.95	1.96	1.92	1.82	1.65	1.39	0.81	0.80
150	0.43	0.65	0.85	1.03	1.20	1.34	1.48	1.60	1.71	1.80	1.88	1.93	1.94	1.90	1.79	1.62	1.37	0.81	0.80
200	0.40	0.61	0.80	0.97	1.13	1.27	1.40	1.51	1.62	1.70	1.77	1.81	1.82	1.78	1.68	1.51	1.28	0.82	0.81
250	0.38	0.57	0.76	0.92	1.07	1.21	1.33	1.44	1.54	1.62	1.68	1.71	1.72	1.68	1.58	1.42	1.20	0.82	0.81
300	0.36	0.54	0.72	0.88	1.02	1.15	1.27	1.37	1.47	1.54	1.60	1.63	1.63	1.59	1.50	1.35	1.13	0.83	0.81
350	0.34	0.52	0.69	0.84	0.98	1.10	1.22	1.32	1.40	1.48	1.53	1.56	1.56	1.52	1.43	1.28	1.08	0.83	0.81
400	0.32	0.49	0.66	0.81	0.94	1.06	1.17	1.27	1.35	1.42	1.47	1.50	1.49	1.45	1.37	1.23	1.03	0.84	0.82
450	0.31	0.47	0.63	0.77	0.90	1.02	1.13	1.22	1.30	1.37	1.42	1.44	1.44	1.40	1.31	1.18	0.99	0.84	0.82
500	0.29	0.45	0.61	0.75	0.87	0.99	1.09	1.18	1.26	1.32	1.37	1.39	1.39	1.35	1.26	1.13	0.95	0.85	0.82
550	0.28	0.44	0.58	0.72	0.84	0.95	1.05	1.14	1.22	1.28	1.32	1.35	1.34	1.30	1.22	1.09	0.91	0.85	0.82
600	0.27	0.42	0.56	0.70	0.82	0.93	1.02	1.11	1.18	1.24	1.28	1.30	1.30	1.26	1.18	1.05	0.88	0.86	0.83
650	0.26	0.41	0.55	0.68	0.79	0.90	0.99	1.08	1.15	1.21	1.25	1.27	1.26	1.22	1.14	1.02	0.85	0.86	0.83
700	0.25	0.39	0.53	0.66	0.77	0.87	0.97	1.05	1.12	1.17	1.21	1.23	1.22	1.19	1.11	0.99	0.83	0.87	0.83
750	0.24	0.38	0.51	0.64	0.75	0.85	0.94	1.02	1.09	1.14	1.18	1.20	1.19	1.15	1.08	0.96	0.80	0.87	0.83
800	0.24	0.37	0.50	0.62	0.73	0.83	0.92	1.00	1.06	1.12	1.15	1.17	1.16	1.12	1.05	0.93	0.78	0.88	0.84
850	0.23	0.36	0.49	0.60	0.71	0.81	0.90	0.97	1.04	1.09	1.12	1.14	1.13	1.09	1.02	0.91	0.76	0.88	0.84
900	0.22	0.35	0.47	0.59	0.70	0.79	0.88	0.95	1.01	1.06	1.10	1.11	1.11	1.07	1.00	0.89	0.74	0.89	0.84
950	0.22	0.34	0.46	0.58	0.68	0.77	0.86	0.93	0.99	1.04	1.08	1.09	1.08	1.04	0.97	0.87	0.72	0.89	0.84
1000	0.21	0.33	0.45	0.56	0.66	0.76	0.84	0.91	0.97	1.02	1.05	1.07	1.06	1.02	0.95	0.85	0.70	0.90	0.85
1250	0.19	0.30	0.41	0.51	0.60	0.69	0.76	0.83	0.88	0.93	0.96	0.97	0.96	0.92	0.86	0.76	0.63	0.92	0.86
1500	0.17	0.27	0.37	0.46	0.55	0.63	0.70	0.76	0.81	0.85	0.88	0.89	0.88	0.85	0.78	0.69	0.58	0.95	0.87
1750	0.15	0.25	0.34	0.43	0.51	0.58	0.65	0.71	0.76	0.80	0.82	0.83	0.82	0.78	0.73	0.64	0.53	0.97	0.88
2000	0.14	0.23	0.32	0.40	0.48	0.55	0.61	0.67	0.71	0.75	0.77	0.77	0.76	0.73	0.68	0.60	0.49	1.00	0.89
2250	0.13	0.22	0.30	0.38	0.45	0.52	0.58	0.63	0.67	0.70	0.72	0.73	0.72	0.69	0.63	0.56	0.46	1.02	0.90
2500	0.13	0.20	0.28	0.35	0.42	0.49	0.55	0.60	0.64	0.67	0.69	0.69	0.68	0.65	0.60	0.53	0.43	1.05	0.92
2750	0.12	0.19	0.27	0.34	0.40	0.46	0.52	0.57	0.61	0.64	0.65	0.66	0.65	0.62	0.57	0.50	0.41	1.07	0.93
3000	0.11	0.18	0.25	0.32	0.38	0.44	0.50	0.54	0.58	0.61	0.62	0.63	0.62	0.59	0.54	0.47	0.39	1.10	0.94
3250	0.11	0.18	0.24	0.31	0.37	0.43	0.48	0.52	0.56	0.58	0.60	0.60	0.59	0.56	0.52	0.45	0.37	1.12	0.95
3500	0.11	0.17	0.23	0.30	0.35	0.41	0.46	0.50	0.54	0.56	0.58	0.58	0.57	0.54	0.49	0.43	0.36	1.15	0.96
3750	0.10	0.16	0.22	0.28	0.34	0.39	0.44	0.48	0.52	0.54	0.55	0.56	0.54	0.52	0.47	0.41	0.34	1.17	0.98
4000	0.10	0.16	0.22	0.27	0.33	0.38	0.43	0.47	0.50	0.52	0.53	0.54	0.52	0.50	0.46	0.40	0.33	1.20	0.99
4250	0.10	0.15	0.21	0.27	0.32	0.37	0.41	0.45	0.48	0.51	0.52	0.52	0.51	0.48	0.44	0.38	0.32	1.22	1.00
4500	0.10	0.15	0.20	0.26	0.31	0.36	0.40	0.44	0.47	0.49	0.50	0.50	0.49	0.46	0.42	0.37	0.30	1.25	1.01
4750	0.10	0.15	0.20	0.25	0.30	0.35	0.39	0.43	0.45	0.48	0.49	0.49	0.47	0.45	0.41	0.36	0.29	1.27	1.03
5000	0.09	0.14	0.19	0.24	0.29	0.34	0.38	0.41	0.44	0.46	0.47	0.47	0.46	0.44	0.40	0.35	0.28	1.30	1.04
5250	0.09	0.14	0.19	0.24	0.28	0.33	0.37	0.40	0.43	0.45	0.46	0.46	0.45	0.42	0.39	0.34	0.28	1.33	1.05
5500	0.09	0.14	0.18	0.23	0.28	0.32	0.36	0.39	0.42	0.44	0.45	0.45	0.44	0.41	0.37	0.33	0.27	1.35	1.06

SKRETTING
a Nutreco company
